# US fathers’ reports of bonding, infant temperament and psychosocial stress based on family sleep arrangements

**DOI:** 10.1093/emph/eoab038

**Published:** 2021-11-17

**Authors:** Lee T Gettler, Patty X Kuo, Mallika S Sarma, Jennifer E Burke Lefever, E Mark Cummings, James J McKenna, Julia M Braungart-Rieker

**Affiliations:** 1 Department of Anthropology, University of Notre Dame, Notre Dame, IN, USA; 2 Eck Institute for Global Health, University of Notre Dame, Notre Dame, IN, USA; 3 William J. Shaw Center for Children and Families, University of Notre Dame, South Bend, IN, USA; 4 Department of Child, Youth and Family Studies, University of Nebraska-Lincoln, Lincoln, NE, USA; 5 Department of Otolaryngology, School of Medicine, Johns Hopkins University, Baltimore, MD, USA; 6 Department of Psychology, University of Notre Dame, Notre Dame, IN, USA; 7 Department of Anthropology, Santa Clara University, Santa Clara, CA, USA; 8 Department of Human Development and Family Studies, Colorado State University, Fort Collins, CO, USA

**Keywords:** cosleeping, bedsharing, solitary sleep, breastsleeping, men's health, paternal care

## Abstract

**Background and objectives:**

Evolutionary-grounded sleep research has been critical to establishing the mutual dependence of breastfeeding and nighttime sleep proximity for mothers and infants. Evolutionary perspectives on cosleeping also often emphasize the emotional motivations for and potential benefits of sleep proximity, including for parent-infant bonding. However, this potential link between infant sleep location and bonding remains understudied for both mothers and fathers. Moreover, in Euro-American contexts bedsharing has been linked to family stress and difficult child temperament, primarily via maternal reports. We know relatively little about whether paternal psychosocial dynamics differ based on family sleep arrangements, despite fathers and other kin often being present in the cosleeping environment across cultures. Here, we aim to help address some of these gaps in knowledge pertaining to fathers and family sleep arrangements.

**Methodology:**

Drawing on a sample of Midwestern U.S. fathers (N=195), we collected sociodemographic and survey data to analyze links between infant nighttime sleep location, paternal psychosocial well-being, father-infant bonding, and infant temperament. From fathers’ reports, families were characterized as routinely solitary sleeping, bedsharing, or roomsharing (without bedsharing).

**Results:**

We found that routinely roomsharing or bedsharing fathers, respectively, reported stronger bonding than solitary sleepers. Bedsharing fathers also reported that their infants had more negative temperaments and also tended to report greater parenting-related stress due to difficulties with their children.

**Conclusions:**

These cross-sectional results help to highlight how a practice with deep phylogenetic and evolutionary history, such as cosleeping, can be variably expressed within communities with the potential for family-dependent benefits or strains.

## Lay Summary

Evolutionary-grounded cosleeping research has elucidated the intimate connections between mother-infant sleep proximity and breastfeeding. However, some Euro-American research indicates that bedsharing can coincide with family strain and stress. Here, U.S. fathers who routinely roomshared or bedshared, respectively, reported stronger bonds to their babies than solitary sleeping fathers, but bedsharing fathers also reported more negative infant temperament.

## INTRODUCTION

Humans’ evolved life history strategy is strongly intertwined with adaptations related to our large, energetically demanding brains, including our propensity to engage in cooperative caregiving to raise our costly young across their protracted dependency period [1–4]. Our developmental trajectory specifically includes a stage of hyper-dependence in infancy, during which human babies experience extensive post-natal brain growth and development and remain fully reliant on caregivers for their survival and safety, while also receiving physiological input and regulation (e.g. respiration, heart rate, sleep state) from caregivers during both daytime and nighttime contact, proximity and care behaviors [5–9]. Evolutionarily, hominin infants would have had their nutritional needs met via breastfeeding during this developmental period. Because human breastmilk is relatively dilute (i.e. low in energy density), it is likely that hominin babies would have typically remained in close proximity to their mothers for frequent feedings across the 24-h day, including during nighttime sleep, as is the case in most other primates [6, 10–12].

Drawing on such cross-species insights as well as cross-cultural perspectives, evolutionary medicine and anthropological research has been foundational to the study of the mutual dependence of human breastfeeding and mother–infant sleep proximity, including on shared sleep surfaces, which is characterized by the recently proposed concept of ‘breastsleeping’ [6, 11, 13, 14]. Given its phylogenetic underpinnings along with the extreme dependency of human infants, the breastsleeping environment is argued to be the sleep ecology to which human babies are adapted evolutionarily [6, 11, 13, 14]. Across human societies, it is also common for fathers and other family members to be present in the cosleeping environment, which is consistent with the importance of cooperative caregiving to the evolution of human life history and converges with sleep ecologies in other primate species that have evolved biparental and alloparental care [6, 11, 14–17]. The present study focuses on infant sleep location and measures of psychosocial and affective dynamics in fathers—an area that has received little attention in the literature [9, 15, 18].

Despite the likely deep evolutionary history of breastsleeping and family sleep proximity in the hominin lineage, dominant cultural traditions in the USA and some Western European societies came to value infant sleep and feeding practices in which formula, bottle fed infants were put to sleep alone in cribs in rooms by themselves [6, 11]. Getting infants to sleep through the night early in the post-partum also became entrenched as a culturally emphasized priority, particularly related to notions of establishing child independence, and, aligning with these values, the parental bed was often viewed as a site for adult (uninterrupted) sleep and conjugal privacy [6, 11, 19–22]. Ultimately, the concomitant prevalence of solitary sleep practices and decrease in breastfeeding in the mid-to-late 20th century in the USA and elsewhere created a potential mismatch between infants’ evolved sleep physiology and the culturally shaped nighttime sleep ecologies to which many were exposed [6, 11, 13, 14]. These mismatched circumstances increased mortality risks for many infants, based on current epidemiological evidence that links solitary sleep and lower breastfeeding duration, respectively, to higher risk of Sudden Infant Death Syndrome [23, 24]. In the last decade, the rates of reported cosleeping, which includes both roomsharing and bedsharing, have risen substantially in the USA, paralleling increased breastfeeding initiation and duration rates in the USA over that time frame [14]. The shifts in breastfeeding and cosleeping behaviors in the USA and elsewhere mean that in two-parent households, fathers and other (non-breastfeeding) parents are also often engaged in or, at least, exposed to a shared sleep environment with their infants. Yet, we know relatively little about how family sleep practices are linked to psychosocial and behavioral profiles in such parents, particularly fathers.

Indeed, the majority of research on this topic focuses solely on mothers and infants, often with an emphasis on breastfeeding pairs [9]. In Euro-American contexts and likely in other settings where families have flexibility in sleeping arrangements, fathers often play a role in joint decision making about family sleep practices in heterosexual couples and, implicitly, in same sex couples with two fathers [15, 25–28]. Nighttime sleep proximity potentially provides an opportunity for additional parent–baby contact and bonding, including for fathers, which is often mentioned as motivation for bedsharing by mothers [9, 29]. This represents a pathway through which cosleeping could be associated with positive functioning for the father–infant relationship. This is generally consistent with evolutionary-grounded perspectives regarding the potential emotional benefits of and motivations for nighttime sleep proximity [30, 31], which, for caregivers could include opportunities for warmth, responsiveness, sensitivity and attunement.

However, the little research that has examined associations between infant sleep location and the quality of parent–child relationships, such as through measures of bonding or attachment, has produced mixed results. For example, in a recent longitudinal study in the UK, mother–infant bedsharing in the first six months post-partum was not linked to variation in later infant–mother attachment or maternal bonding [32]. Meanwhile, research in the Netherlands found that infants who slept solitarily at 2 months of age were more likely to be insecurely attached at 14 months, compared to infants who had bedshared [33]. Prior work has not explored father–infant bonds in relationship to infant sleep location, to our knowledge.

In cultural settings with meaningful variation in family sleep practices, e.g. solitary versus cosleeping, enhanced opportunities for father–infant bonding via nighttime proximity could feedback to greater daytime paternal care. In addition, cross-cultural research on paternal psychobiology has found that fathers’ who cosleep have lower testosterone, on average, than fathers who sleep separate from their children [34, 35]. Lower testosterone in parents is argued to facilitate nurturance and has been linked to fathers’ greater participation in daily childcare in a range of cultures [36, 37]. Thus, there are plausible pathways through which covariation between sleep practices and daytime paternal care could occur. However, in two studies, to date, that tested for such a link, solitary sleeping versus cosleeping Filipino fathers did not significantly vary for their amount of daily childcare [34], and US fathers’ percentage of time as their child’s primary caregiver also did not significantly differ based on sleep practices [22]. Elsewhere, in a study that followed Israeli families longitudinally when infants were 3–18 months old, fathers in roomsharing families were less involved with both overall caregiving and nighttime care across the study period compared to fathers in solitary sleeping families [38].

Moreover, some US-based studies have shown that cosleeping is associated with negative attitudes about the coparenting relationship [26], of which division of childcare labor is a component. As argued by Mileva-Seitz *et al*. [18], bedsharing may be linked to parental stress and marital distress in cultural contexts like the USA where bedsharing has historically been perceived negatively, even if recently such beliefs have attenuated somewhat in segments of the population. This may be especially true if partners disagree about family sleep arrangements or if bedsharing is ‘reactive’ in response to infant/child night wakings [22, 28, 39], which may likewise be seen as problematic nighttime behavior in some families that emphasize the importance of early sleep consolidation [18]. Along these lines, research in Euro-American societies has shown that cosleeping was associated with parental reports about challenging infant temperament and strain between partners [26, 39–42]. Overall, associations between cosleeping and paternal stress could be linked to negative functioning of the family system, including fathers’ coparenting and bonding.

To help contribute to the relatively small literature on fathering and infant sleep arrangements, we combine data from two studies that we conducted in the US Midwest (*N* = 195) to test for links between infant sleep location and fathers’ reports of bonding, paternal stress, negative infant affect and involvement in daily caregiving. We predicted that fathers who slept in closer proximity (roomsharing or bedsharing, respectively) would report stronger bonding to their infants than those with solitary sleeping infants. Given prior results [26, 38, 40–42], we also test whether infant sleep location was linked to paternal reports of psychosocial stress, negative infant temperament and fathers’ caregiving involvement.

## METHODS

### Study design and participants

We draw on two studies conducted in the South Bend, IN metropolitan area between 2013 and 2016. In study 1 (S1), we recruited families with infants between 5 and 7 months of age (*N* = 47; mean infant age: 6.12 months, 1.25 SD) for participation in a lab-focused study on parent–infant interaction and family well-being. In study 2 (S2), we recruited fathers whose babies were born at a local hospital for a study focused on psychobiology around birth and fathers’ parenting and well-being 2–4 months later (*N* = 148; mean infant age: 2.80 months, 1.68 SD). In the pooled analyses, fathers were 31.97 years old (5.56 SD) and predominantly white (∼84%), with ∼45% having less than a 4-year college degree. We report further descriptive statistics and bivariate correlations for key study variables in Tables 1 and 2 and Supplementary Table S1, which includes demographic data separated by study (S1 vs S2). One father from S1 was excluded based on his child being >17 months old. The Institutional Review Boards at the University of Notre Dame and Memorial Hospital approved all procedures for the study and all participants provided written informed consent.

**Table 1.** Descriptive statistics and bivariate correlations (*r*) between key continuous study variables^a^^,^^b^

	1	2	3	4	5	6	7	8	9	10	Mean	SD	*N*
1. Infant age (months)	1.00 (195)										3.63	2.19	195
2. Father age (years)	0.14 (184)	1.00 (184)									31.97	5.56	184
3. Bonding score	-0.05 (139)	-0.02 (128)	1.00 (139)								79.32	9.85	139
4. PSI total score	0.09 (187)	-0.14 (177)	-0.55*** (137)	1.00 (187)							62.41	16.06	187
5. PSI difficult child	0.11 (187)	-0.13 (177)	-0.46*** (137)	0.84*** (187)	1.00 (187)						20.57	6.28	187
6. PSI parent distress	0.14 (187)	-0.08 (177)	-0.48*** (137)	0.85*** (187)	0.52*** (187)	1.00 (187)					24.12	7.57	187
7. IBQ neg. affect	0.21** (184)	0.03 (174)	-0.24** (134)	0.37*** (180)	0.37*** (180)	0.33*** (180)	1.00 (184)				3.43	0.93	184
8. CCAS direct care (%)	0.14* (195)	-0.24** (184)	0.23** (139)	-0.12 (187)	-0.13 (187)	-0.05 (187)	-0.13 (184)	1.00 (195)			25.47	13.53	195
9. CCAS indirect care (%)	0.04 (195)	-0.13 (184)	0.19* (139)	-0.12 (187)	-0.17* (187)	0.01 (187)	-0.05 (184)	0.42*** (195)	1.00 (195)		27.02	14.77	195
10. CCAS play (%)	0.10 (195)	-0.15* (184)	0.33*** (139)	-0.21** (187)	-0.21** (187)	-0.12 (187)	-0.09 (184)	0.45*** (195)	0.48*** (195)	1.00 (195)	36.24	12.74	195

a Pearson’s *r* is listed in the top row of each cell with the bivariate sample size in parentheses in the second row.

b Survey data came from the following instruments: Bonding score, Paternal–Infant Attachment Scale; PSI scores, Parenting Stress Index; IBQ neg. affect, Infant Behavior Questionnaire; CCAS scores, Childcare Activities Scale. See the Methods for further details and references.

* *P* < 0.05,

** *P* < 0.01,

*** *P* < 0.001.

**Table 2.** Additional descriptive statistics for non-continuous study variables

Family sleep practices	
Solitary sleepers (% yes)	29.7
Roomsharers (% yes)	50.3
Bedsharers (% yes)	20.0
Sociodemographics	
Infant currently breastfeeding or receiving breastmilk (% yes)^a^	75.9
Experienced father (% yes)	50.3
Fathers' education level	
Less than 4-year college degree (%)	45.2
4-Year college degree or more (%)	54.8
Race and ethnicity	
Black/African American (%)	5.6
Hispanic (%)	5.1
Other race/ethnicity (%)	5.1
White (%)	84.1

a This variable potentially includes some infants who were receiving breastmilk from a bottle.

### Survey instruments

At home (S1, S2) and during lab visits (S1), fathers filled out validated surveys, including the Parenting Stress Index (PSI-SF), which is a 36-item instrument that provides a total overall score for psychosocial stress related to family life (PSI total: Cronbach’s alpha 0.93) as well as sub-scores for ‘PSI parental distress’ (e.g. ‘I feel trapped by my responsibilities as a parent’, alpha 0.85) and ‘PSI difficult child stress’ (e.g. ‘I feel that my child is very moody and easily upset’, alpha 0.86) [43]. Fathers filled out the Infant Behavior Questionnaire, which describes aspects of infant development and temperament. We focus on the ‘IBQ negative affect’ sub-scale (e.g. ‘At the end of an exciting day, how often did your baby become tearful?’, alpha 0.80) [44]. Fathers reported their involvement in childcare from 0% to 100% in three domains using the Childcare Activities Scale (CCAS): direct care (e.g. ‘bathing child’, alpha 0.71), indirect care (e.g. ‘arranging babysitting’, alpha 0.57) and play (e.g. ‘playing actively with child’, alpha 0.80) [45]. The CCAS also includes a single item pertaining to nighttime care (i.e. ‘going to child at night if child awakens’), which we also analyzed independently, given the focus of the present analyses and prior results elsewhere [38].

Finally, in S2 only, fathers reported on their bonding with their infants through the Paternal–Infant Attachment Scale (alpha = 0.86). For example, fathers are asked, ‘Over the last two weeks I would describe my feelings for the baby as’, with responses from ‘dislike’ to ‘intense affection,’ and ‘I can understand what my baby needs or wants,’ with responses from ‘almost never’ to ‘almost always’ [46]. We also note that not all men in each study completed the full breadth of surveys, as some participants elected to not answer certain questions in some surveys and, in other cases, chose not to complete specific surveys entirely. Thus, the sample sizes vary across analyses based on specific instruments/domains, as reported in Tables 1 and 3.

**Table 3.** Regression models predicting fathers’ post-partum bonding, infant temperament, psychosocial stress and caregiving involvement^a^^,^^b^

	Father–infant bonding (*N* = 139)			IBQ infant negative affect (*N* = 184)		
	*b*	95% CI	*p*	*b*	95% CI	*p*
Roomsharing	0.61	(0.20, 1.03)	0.004	0.13	(−0.22, 0.49)	0.468
Bedsharing	0.60	(0.08, 1.13)	0.024	0.49	(0.07, 0.90)	0.023
Child age (*z* score)	−0.02	(−0.24, 0.20)	0.837	0.22	(0.06, 0.37)	0.006
Experienced father	−0.39	(−0.71, −0.06)	0.020	0.06	(−0.23, 0.35)	0.698
Breastfeeding^c^	−0.30	(−0.70, 0.10)	0.145	−0.01	(−0.35, 0.33)	0.963
Model R2		0.11			0.08	

	PSI total stress (*N* = 187)			PSI parent distress (*N* = 187)		
		
	*b*	95% CI	*p*	*b*	95% CI	*p*

Roomsharing	0.09	(−0.27, 0.44)	0.639	−0.07	(−0.43, 0.29)	0.707
Bedsharing	0.32	(−0.10, 0.74)	0.129	0.19	(−0.23, 0.61)	0.373
Child age (*z* score)	0.11	(−0.05, 0.27)	0.165	0.12	(−0.03, 0.28)	0.119
Experienced father	−0.13	(−0.42, 0.16)	0.383	−0.11	(−0.40, 0.18)	0.443
Breastfeeding^c^	0.18	(−0.17, 0.52)	0.315	0.03	(−0.31, 0.38)	0.843
Model R2		0.03			0.03	

	PSI difficult child stress (*N* = 187)^d^			CCAS direct care (*N* = 195)		
		
	*IRR*	95% CI	*p*	*b*	95% CI	*p*

Roomsharing	1.06	(0.95, 1.18)	0.272	−0.16	(−0.49, 0.16)	0.324
Bedsharing	1.12	(0.99, 1.27)	0.067	−0.05	(−0.43, 0.33)	0.792
Child age (*z* score)	1.05	(1.00, 1.09)	0.048	0.10	(−0.04, 0.25)	0.151
Experienced father	0.99	(0.91, 1.08)	0.819	−0.49	(−0.75, −0.23)	0.001
Breastfeeding^c^	1.07	(0.97, 1.18)	0.202	−0.69	(−1.00, −0.38)	0.001
Model R2		–			0.18	

	CCAS indirect care (*N* = 195)			CCAS play (*N* = 195)		
		
	*b*	95% CI	*p*	*b*	95% CI	*p*

Roomsharing	0.15	(−0.19, 0.50)	0.384	0.27	(−0.08, 0.62)	0.123
Bedsharing	0.12	(−0.29, 0.53)	0.552	−0.02	(−0.43, 0.39)	0.929
Child age (*z* score)	0.14	(−0.01, 0.29)	0.066	0.15	(−0.01, 0.30)	0.061
Experienced father	−0.13	(−0.41, 0.15)	0.366	−0.34	(−0.62, −0.06)	0.019
Breastfeeding^c^	−0.48	(−0.81, −0.14)	0.005	−0.24	(−0.58, 0.09)	0.152
Model R2		0.07			0.07	

a All models are from OLS regression analyses with dependent variables in SD units, except for PSI difficult child. This model was analyzed with negative binomial regression with a dependent variable that was not converted to SD units. Comparison groups for categorical variables: solitary sleeping fathers; first-time fathers; babies not currently breastfeeding and not otherwise receiving breastmilk.

b Survey data came from the following instruments: Bonding score, Paternal–Infant Attachment Scale; PSI scores, Parenting Stress Index; IBQ neg. affect, Infant Behavior Questionnaire; CCAS scores, Childcare Activities Scale. See the Methods for further details and references.

c This variable potentially includes some infants who were receiving breastmilk from a bottle in the ‘still breastfeeding’ category.

d Cohen’s *d* comparing PSI difficult child stress for: bedsharing versus solitary sleeping fathers, *d* = 0.36; roomsharing versus solitary sleeping fathers, *d* = 0.09.

### Sleep practices

In the pooled data from both studies, fathers were characterized as solitary sleepers (29.7%), roomsharers (50.3%) and bedsharers (20.0%) (Table 1). In S1, fathers reported how many nights per week they slept in the same room or same bed as their infants in sequential questions. Men could characterize themselves as both roomsharers and bedsharers (i.e. they were not mutually exclusive), but we defined routine bedsharers as those reporting >3 nights of bedsharing and routine roomsharers as those with >3 nights of roomsharing (without bedsharing). Fathers first reported roomsharing in response to the question, ‘About how many nights per week do you sleep in the same room as your child(ren)?’ Of the fathers with full data for the current analyses in study 1 (*N* = 47), 44.7% reported not sharing a room with their infants for any night per week, 19.0% reported roomsharing 1–3 nights per week, and the remaining 36% reported roomsharing 4–7 nights per week.

Fathers then responded to the question, ‘About how many nights per week do you bring your baby (5–7 month old) to sleep with you in your bed? (This could be for any amount of time, even a few minutes).’ A majority of S1 fathers, 53.2%, reported never bedsharing during the week, while 17.0% reported bedsharing between 1 and 3 nights per week, and 29.8% reported bedsharing 4 and 7 nights per week. Because the roomsharing and bedsharing questions were separate and not mutually exclusive, fathers could have reported a small number of nights (i.e. <4 nights) for each arrangement as distinct, thereby leading to their miscategorization as solitary sleepers in our study. However, this definitional concern only applied to one father who we categorized as a solitary sleeper. He reported two nights of roomsharing and two nights of bedsharing, with the latter being potentially, if not likely, subsumed under the umbrella of the former.

In S2 (*N* = 148), fathers responded to the multiple-choice question, ‘Where does your baby sleep at night?’ Fathers responded to the following options, with the percent who chose that option in parentheses: in a crib or bassinet in a separate room (21.6%), in a crib or bassinet in my room (23.7%), in a crib or bassinet next to my bed (36.5%), in my bed with me (2.0%), sometimes with mother or me in bed, sometimes in his or her own crib (14.2%) and other (2.0%). The individuals reporting ‘other’ provided verbatim reports that aligned with roomsharing (*N* = 2) or bedsharing (*N* = 1).

### Breastfeeding data

In S1, mothers reported whether they were currently breastfeeding or not as well as on the frequency of breastfeeding across the 24-h day. However, they did not report whether their infants were receiving other foods, precluding us from characterizing infants as exclusively breastfed in S1. Mothers reported that 68.1% of S1 infants were still breastfeeding (Supplementary Table S1). In S2, fathers responded to the question ‘How is your baby fed?’ with the following options: using a bottle (formula only), using a bottle (formula mixed with cereal), breastfeed only (including breast milk in a bottle), and breastfeed and bottle feed. In S2, 78.4% of infants were still receiving breast milk (Supplementary Table S1), while 52% were exclusively breastfed.

### Statistical analyses

We conducted all analyses with Stata v. 14.0. For descriptive purposes, we began by analyzing bivariate correlations between key continuous study variables (Table 1). In our core models, we primarily used ordinary least squares regression with the exception of the model for fathers’ PSI difficult child stress, as this variable was over dispersed. Consequently, we used negative binomial regression for modeling this measure as a dependent variable. For this measure (PSI difficult child), we also report Cohen’s *d* comparisons between fathers for solitary sleeping versus roomsharing or bedsharing, respectively, to provide comparable effect sizes to the OLS regression models in which we converted the dependent variables to SD units (*z* scores).

We included covariates that could potentially confound links between sleep practices and our measures, including: infant age, infant feeding method, paternal experience and paternal education. We note that a sub-sample of otherwise eligible men (*N* = 7) did not report their educational attainment. We evaluated whether paternal educational attainment increased model fit using Akaike information criterion (AIC). The inclusion of this covariate did not improve the model fit for any analyses except for the model for direct caregiving and that model improvement was modest (AIC: 485.1 without educational attainment; AIC: 484.8 with educational attainment). We did not include this variable in our finals models, which we report in Supplementary Table S3.

Finally, based on our prior findings from S2 [47] and following the results in Supplementary Table S3, we conducted a post-hoc analysis to assess whether the links between infant sleep arrangements and father–infant bonding were independent of fathers’ daytime caregiving involvement.

## RESULTS

We report descriptive statistics for the study participants in Tables 1 and 2, along with bivariate correlations between key continuous variables in Table 1. In addition, we also include demographic statistics separate by study (S1 vs S2) in Supplementary Table S1.

**Figure 1.**
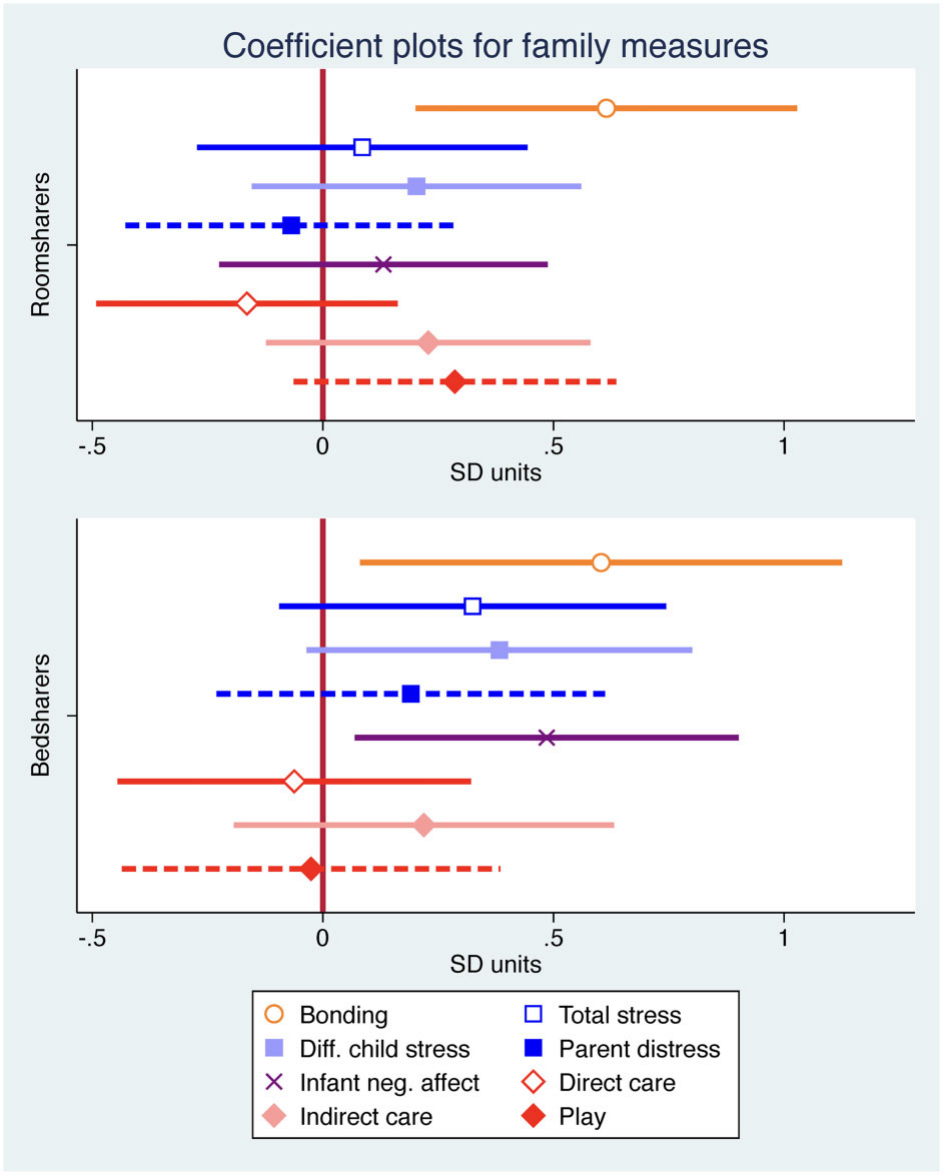
Coefficient plots following OLS regression models for infant sleep arrangements predicting fathers’ reports of bonding, psychosocial stress, infant negative (neg.) affect and daytime father involvement in childcare, adjusting for covariates. All of the variables are in SD units. The plotted coefficients reflect the models in Table 3, with the exception of Diff. child stress, which was formally analyzed with negative binomial regression. These models include the following covariates: child age, paternal experience and infant breastfeeding status (see Table 3)

Compared to solitary sleeping fathers, roomsharing and bedsharing fathers reported significantly stronger bonds to their infants (both *P* < 0.05; Table 3; Fig. 1). However, bedsharing fathers reported higher levels of negative infant temperament (*P* = 0.023; Table 3) and modestly greater difficult-child stress than solitary sleeping fathers, though the difference was not statistically significant (*P* = 0.067; Cohen’s *d* = 0.36; Fig. 1). There were no significant differences between the groups for any other outcome. In addition to the childcare measures in Table 3, fathers did not differ for their percentage involvement in caring for children during night wakings (Neg. binomial reg: both *P* > 0.9), with similar reports across solitary sleeping (24%), roomsharing (22.2%) and bedsharing (23.5%) fathers. The effect sizes and CIs for our core analyses are visually represented in Fig. 1 and reported in Table 3.

Given our prior results linking fathers’ daytime caregiving to fathers’ bonding [47] and the analogous bivariate pattern here in Table 1, with all three measures of care being positively correlated to fathers’ bonding scores (*r*s = 0.19–0.33), we conducted a post-hoc analysis to assess whether the links between infant sleep location and father–infant bonding were independent of fathers’ overall caregiving involvement. In that model, the effect size for bonding comparisons between roomsharing and solitary fathers was minimally changed (*b*, 95% CI: 0.60, 0.20–1.01; *P* = 0.003). Meanwhile the effect size comparing bonding scores for bedsharing fathers versus solitary sleepers increased (*b*, 95% CI: 0.71, 0.20–1.22; *P* = 0.006). This suggests that overall caregiving involvement somewhat masked the association between bedsharing and bonding, in comparison to solitary sleeping, rather than overall caregiving confounding the relationship.

## DISCUSSION

In this cross-sectional analysis of infant sleep arrangements and US fathers’ reports of affective and behavioral dynamics within the family, pertinent to evolutionary perspectives on shared family sleep environments, we found that fathers who routinely roomshared or bedshared with their infants reported having stronger bonds with them. The medium-level effect sizes were similar for bonding comparisons between roomsharing or bedsharing fathers, respectively, and solitary sleepers. To our knowledge, this is one of the first studies to focus on the quality of parent–infant bonds in relationship to family sleeping arrangements, particularly for fathers [9]. These patterns linking closer nighttime sleep proximity to stronger self-reported bonding for fathers to infants are consistent with evolutionary-grounded perspectives on cosleeping. Such framing often includes an emphasis on parental emotional motivations for warmth, responsiveness, sensitivity and physical touch that can be afforded by cosleeping, which conceptually could contribute to and be enhanced by fathers’ bonds to their infants [30, 31]. In contrast, in a UK-based study, mothers’ reported bonds to their infants did not significantly differ based on mother–infant bedsharing during infancy [32]. While diverging with this latter study, our findings generally align with past research on family motivations to bedshare, in which parents (typically mothers) from diverse racial/ethnic and cultural backgrounds often emphasized the importance of nighttime proximity for providing emotional comfort, security and bonding as well as affording fathers more time with the baby [9, 29].

Yet, we also found that fathers’ reported involvement in nighttime care in response to child wakings did not differ by infant sleep location. Fathers engaged in a relatively low percentage of nighttime care (mean: 23.0%), compared to mothers (∼76.4%), which is generally consistent with past US research showing that mothers disproportionately bear the responsibility for nighttime care of infants and young children [48, 49]. Similarly, fathers’ overall involvement in multiple domains of routine caregiving was also comparable across infant sleeping arrangements. This is consistent with the idea that family sleep practices and patterns of shared daytime caregiving between parents are likely to be largely independent of one another, at least in this study population. This aligns with two past studies that have failed to find links between fathers’ daily caregiving and family sleep arrangements in the USA and the Philippines, respectively [22, 34], although roomsharing fathers were found to be less involved in care (overall and during nighttime) relative to solitary sleeping fathers in Israel [38]. Here, our results also indicate that cosleeping fathers’ higher bonding scores were not explained or confounded by overall caregiving involvement, which we confirmed in a post-hoc analysis.

However, contrary to the broader, potentially beneficial implications of nighttime parent–infant sleep proximity often emphasized from an evolutionary perspective [30, 31], we also found that bedsharing fathers reported higher levels of negative infant temperament and tended to report greater difficult-child stress compared to solitary sleepers. These patterns are notable given prior US-based research showing that mothers reported a greater likelihood of bedsharing/cosleeping under conditions of ‘partner strain’ (i.e. stress related to the father) [40] and maternal marital distress [26]; US families were also more likely to cosleep with children perceived to have difficult temperaments [9]. These results contrast with our finding that bedsharing fathers reported greater father–infant bonding. Father–infant bonding scores were significantly negatively correlated with both measures (difficult-child stress, *r* = −0.47; negative temperament, *r* = −0.24) in our sample. This is consistent with the notion that the benefits or strains of sleep practices are likely to be family dependent. Specifically, Euro-American research indicates that reactive cosleeping (i.e. in response to sleep difficulties) is linked to more negative outcomes than is routine, planned cosleeping with mutual parental commitment to the practice [9, 18].

Our lack of data in this domain (reactive vs planned cosleeping) represents one limitation of the present analyses, as we are not able to test whether such family characteristics help to better clarify the somewhat conflicting results we found for bonding and stress/temperament [9, 18]. Because our data on infant sleep arrangements are self-report, it is also possible that the frequency of bedsharing was underreported, given the practice continues to be frowned upon and often discouraged as well as stigmatized in the USA [50, 51], including in comparison to roomsharing, which is recommended by the American Academy of Pediatrics [14]. Moreover, in the present analyses, we combined data from two studies in which infant sleep arrangements were collected through two different approaches. For S1, it is possible that fathers could be miscategorized because roomsharing and bedsharing were not mutually exclusive. However, this only applied to a single S1 father, as we noted in the Methods. Importantly, if fathers underreported bedsharing in either study and identified as solitary sleepers or roomsharers this could limit our ability to detect significant differences between groups (i.e. Type II error); this issue would not increase our likelihood of false positives (Type I error), including for the core findings in the present analyses. A final limitation is the cross-sectional research design, which prevents us from testing for temporal relationships between variables, such as whether sleep practices early in the post-partum predict changes in outcomes such as bonding and parental stress over the infancy period.

In conclusion, in a sample of US fathers living in the Midwest, we found that those who slept in closer proximity to their infants at night via roomsharing or bedsharing reported feeling more bonded to their babies. However, our results also show indications that bedsharing is linked to paternal perceptions of negative infant temperament and fathers’ reports of parenting stress pertaining to difficulties with their child. These latter findings do not necessarily align with evolutionary framing on potential benefits and selective advantages (in the past) of nighttime sleep proximity, but are more consistent with biocultural perspectives in this area, which emphasize the historically situated and culturally contextualized expression of such practices, alongside their evolutionary history [6, 11, 14, 50]. We hope these cross-sectional results help to further stimulate research on the potential benefits and costs of diverse sleep practices to family members in addition to mothers and infants. Such work can enhance our understanding of how practices that likely have deep evolutionary and phylogenetic histories, such as breastsleeping and family sleep proximity, come to be expressed in contemporary societies and individual families, with a range of culturally grounded values and norms that may shape the health-related outcomes for infants and caregivers.

## Supplementary Material

eoab038_Supplementary_Data
