# Supplementary material for: US fathers’ reports of bonding, infant temperament and psychosocial stress based on family sleep arrangements
Source: Evol Med Public Health. 2021 Nov 17;9(1):460–9. doi: 10.1093/emph/eoab038 (PMC8830308; doi:10.1093/emph/eoab038)
Supplement: eoab038_Supplementary_Data [file eoab038_Supplementary_Data.docx]

| **Supp. Table 1. Descriptive statistics by study** | | | | | | |
| --- | --- | --- | --- | --- | --- | --- |
|  | *Study 1* | |  | | *Study 2* | |
|  | Mean | SD |  | Mean | | SD |
| *Family sleep practices* |  |  |  |  | |  |
| Solitary sleepers (% yes) | 55.3 |  |  | 21.6 | |  |
| Roomsharers (% yes) | 14.9 |  |  | 61.5 | |  |
| Bedsharers (% yes) | 29.8 |  |  | 16.7 | |  |
| *Sociodemographics* |  |  |  |  | |  |
| Infant age (months) | 6.11 | 5.72 |  | 2.84 | | 1.80 |
| Father age (years) | 33.13 | 5.72 |  | 31.58 | | 5.47 |
| Infant currently breastfeeding (% yes)^a^ | 68.1 |  |  | 78.4 | |  |
| Experienced father (% yes) | 51.1 |  |  | 50.0 | |  |
| Fathers' education level |  |  |  |  | |  |
| Less than 4-year college degree (%) | 28.3 |  |  | 50.7 | |  |
| 4-year college degree or more (%) | 71.7 |  |  | 49.3 | |  |
| *Race and Ethnicity* |  |  |  |  | |  |
| Black/African American (%) | 4.3 |  |  | 6.1 | |  |
| Hispanic (%) | 8.5 |  |  | 4.1 | |  |
| Other race/ethnicity (%) | 8.5 |  |  | 4.1 | |  |
| White (%) | 78.7 |  |  | 85.8 | |  |
|  |  |  |  |  | |  |

^a^This variable potentially includes some infants who were receiving breastmilk from a bottle.
